# Understanding error culture in veterinary medicine: a survey among veterinarians across German-speaking countries

**DOI:** 10.3389/fvets.2026.1784869

**Published:** 2026-04-07

**Authors:** Corinna M. Montag, Christin Kleinsorgen, Dana Rösch-Rodax, Holger A. Volk, Claudia Busse

**Affiliations:** 1 Department of Small Animals Medicine and Surgery, University of Veterinary Medicine Hannover, Hannover, Germany; 2 Centre for Teaching, E-Learning-Services, University of Veterinary Medicine Hannover, Hannover, Germany; 3 Outpatient Institute Clinic Psychotherapy, Johannes Gutenberg University, Mainz, Germany

**Keywords:** error reporting, just culture, medical error culture, error management, veterinary practice

## Abstract

**Introduction:**

Errors are assumed to occur frequently in veterinary practice and may affect animal health, client trust, and the well-being of veterinary staff. Their handling is shaped by the prevailing error culture within practices and institutions. While error culture has been investigated in human medicine and extensively in other high-risk fields such as aviation, little is known about how it is perceived within veterinary medicine

**Methods:**

This study therefore examined practising veterinarians’ perceptions of errors, contributing factors, reporting practices, and institutional responses. An anonymous online questionnaire, adapted from a British pilot study and expanded to 29 items, collected demographic data, experiences with errors, contributing factors, and institutional structures. Three perspectives were captured: self-reported errors, errors reported to supervisors, and errors observed within the team

**Results:**

A total of 1102 fully completed questionnaires were analysed. More than three-quarters of participants (*n* = 858, 78%) reported at least one incident in which an animal suffered permanent harm or death, and 68% (*n* = 745) stated that they had disclosed at least one of their own. Based on the weighted ranking, diagnostic activities were ranked first, followed by medication dosing and surgical procedures among self-reported errors. Supervisors most strongly prioritized interaction with animal owners, followed by billing and the diagnostic activities in relation to error reports received. Similarly, observed errors were most strongly prioritized in relation to interactions with animal owners, followed by diagnostic activities and medication dosing. Across all perspectives, the same central contributing factors were identified: time pressure, a hectic working environment, and lack of experience. Reasons for not reporting errors included the belief that the incident was irrelevant (*n* = 130, 53%) or the perception that no errors had occurred (*n* = 46, 19%). More than half of respondents reported no formal error reporting system existed in their workplace (*n* = 579, 54%); errors were mainly discussed in one-to-one conversations (*n* = 821, 75%). Only 40 respondents (4%) reported the presence of anonymous reporting systems, whereas 392 respondents (36%) reported dedicated team meetings.

**Discussion:**

Overall, although errors are common, the organizational conditions required for systematic identification, communication, and learning are lacking in many settings.

## 1 Introduction

The concept of “error” originates from the Latin verb “errare,” meaning “to stray” or “to wander,” representing a deviation from an intended outcome (1). In modern usage, particularly in high-stakes domains such as healthcare and aviation, “error” refers to failures in judgment or execution that lead to unintended outcomes, arising from normal cognitive processes rather than from negligence or incompetence. The development of “Error Culture,” specifically “Just Culture,” arose from recognizing that errors should be opportunities for improvement rather than punishment (2). The term “Error Culture” describes how organizations or professional groups deal with errors. “Just Culture” is a specific approach within this framework that distinguishes between unintentional human errors, risky behavior, and deliberate rule violations, and focuses on learning from errors rather than blaming individuals for honest mistakes (3). Over time, organizations saw that a culture allowing employees to discuss and learn from errors led to better performance and innovation. For example, the Aviation Safety Reporting System (ASRS) encourages error reporting without fear of retaliation (4). In veterinary medicine, issues of error management are likewise gaining importance. A Europe-wide study from 2022, analyzing 2,155 incident reports from small animal clinics, identified medication errors, communication problems, and organizational weaknesses as the most common causes of incidents. A large proportion of these events were potentially preventable, highlighting a considerable need for improvement in error prevention and internal safety management within veterinary practices (5, 6). In this context, errors in veterinary practice often arise from the interaction between individual actions and organizational conditions. Therefore, in the present study, the term “error” was applied in a broad sense, encompassing both individual actions and systemic causes, in line with contemporary concepts of Error Culture and Just Culture (2). Establishing a Just Culture can play an important role in encouraging error reporting, fostering transparency, and addressing underlying factors such as fatigue, stress, and communication breakdowns. Models such as Heinrich’s Domino Theory and Reason’s Swiss Cheese Model illustrate how individual actions and systemic conditions interact in complex working environments to create the potential for errors, and both models can be applied to veterinary practices (7). This concept was formally incorporated into aviation safety regulation by the International Civil Aviation Organization (ICAO) in 2005, building on earlier theoretical work on human error and system safety, most notably by James Reason (8).

In aviation, flight safety depends on the collective commitment of aircraft crew and management (9). This joint approach, involving individual professionals as well as organizational leadership, finds its parallel in healthcare, where a strong safety culture is crucial for improving patient outcomes. In human medicine, various tools and systems, such as voluntary incident reporting schemes, Critical Incident Reporting Systems (CIRS), and safety checklists (e.g., the “WHO Safe Surgery Checklist”), have proven effective in reducing errors (10, 11). In addition, methods such as Root Cause Analysis (RCA) and programs like TeamSTEPPS promote teamwork and support the systematic prevention of errors (12, 13).

Veterinary medicine, by contrast, is still at an early stage in developing a systematic approach to error management. This assessment is supported by previous publications indicating that, compared with human healthcare, veterinary medicine lacks widely established patient safety frameworks, structured incident reporting systems, and systematic research on safety culture and error management (6, 14). In that context, the purpose of the present study was to explore veterinarians’ subjective perceptions and experiences regarding how errors are addressed and discussed in veterinary practice within German-speaking countries. The focus was on error reporting, the areas in which errors occur, and the factors contributing to their occurrence. To capture these aspects, different perspectives and their influence on the overall error culture within veterinary institutions were considered. Furthermore, the study explored how errors are discussed and managed in everyday clinical practice. By incorporating both individual and organizational perspectives, the developed questionnaire was designed to provide a comprehensive assessment of existing structures and approaches to handling errors.

## 2 Methods

### 2.1 Development of the questionnaire

The questionnaire used in this study was developed based on a previously published survey that examined errors made by recently graduated veterinarians in the United Kingdom (15). In total, 16 of the original questions were revised, and the survey conducted in this study comprised 29 questions. The term error was used throughout the questionnaire instead of mistake, as it is the established terminology in healthcare research and more accurately reflects clinical, procedural, and systemic aspects relevant to veterinary practice. In the survey, the term error was not explicitly defined but was used openly to capture the individual experiences of participants. This approach was consistent with other studies, in which errors are understood as events with potential or actual negative effects on patients (16).

### 2.2 Content structure and design

The questionnaire was divided into four thematic main sections.

The first section addressed the demographic data, including the duration of the participants’ professional veterinary activity (time since graduation). This information was collected using single-choice questions and served to categorize respondents according to their level of professional qualification and experience. The variables age (entered as a year of birth), gender, number of employees at the workplace, average number of patients per day, level of professional training, type of workplace, and employment status were surveyed. Depending on the variable, single- or multiple-choice formats were applied to allow both distinct categorization and multiple responses where appropriate. This section contained professional and demographic variables to characterize the composition of the study population.

The second section focused on the topic of error reporting. This part aimed to explore whether errors are reported, as well as to identify where errors occur and which factors contribute to them within veterinary practice. Three perspectives were considered: self-reported errors, errors received as supervisors and observed error reports. For each of these groups, a set of analogous questions was used to ensure comparability across perspectives. Participants were asked to indicate the types of activities in which, in their perception, errors most commonly occurred (ranking scale) and which factors most frequently contributed to such errors (multiple choice). Additionally, participants could indicate if they had not reported, received, or observed any errors to avoid bias through forced responses.

The third section addressed the handling of errors within the team, including feedback and institutional reporting systems. Participants were first asked to evaluate how error reporting is handled in their own workplace. They were then asked whether a formal reporting system existed and in what form post-error discussions typically occurred. These questions were presented in single- and multiple-choice formats. The evaluated aspects included the reporting system itself, the discussion of errors, the availability of time and material resources for error prevention, the level of support from supervisors and veterinary medical staff, and the degree of moral support within the institution.

The fourth section consisted of an open-ended question inviting participants to share additional reflections, experiences, or suggestions related to error culture in veterinary practice, thereby capturing qualitative insights that could not be obtained through structured response formats.

This survey design and structure enabled a comprehensive analysis of error culture in everyday veterinary practice by integrating both individual and organizational dimensions.

### 2.3 Sample notation

To improve clarity in the presentation of results, different subscripts are used to distinguish respondent subgroups. *N* refers to the total number of fully completed questionnaires (*N* = 1,102). *n* denotes the number of respondents to a specific item. The subgroup *n_s__1_* comprises all participants with supervisory responsibilities, while *n_s2_* refers to supervisors who had received at least one error report. The subgroup *n_o__1_* includes all participants who had observed an error notification, and *n_o__2_* refers to those who were able to identify contributing factors to the observed errors. These notations are applied consistently throughout the results section.

### 2.4 Pretest and revision

A preliminary version of the questionnaire was piloted with 31 veterinarians at the Small Animal Clinic of the University of Veterinary Medicine Hannover. Participants evaluated the clarity, relevance, structure, and usability of the questions. Based on this feedback, the questionnaire was subsequently revised by shortening several questions, simplifying the response options, and removing redundant questions to reduce respondent burden and minimize premature survey dropout. These revisions resulted in the final version of the questionnaire.

### 2.5 Study population and inclusion criteria

Eligible participants were licensed veterinarians who were actively working in clinical practice at the time of data collection. Individuals from German-speaking regions, including Germany, Austria, and Switzerland, were included. Other professional groups or students were excluded from participation.

### 2.6 Data collection and ethical considerations

The final version of the online questionnaire was implemented anonymously using LimeSurvey (Community Edition Version 6.13.2 + 250,506, LimeSurvey GmbH, Hamburg, Germany) and was available between May 22 and July 31, 2024. The survey was distributed via the social media channels of the University of Veterinary Medicine Hannover and through various professional mailing lists and veterinary organizations, including the TVD-Finanz GmbH & Co. KG database, the German Veterinary Chambers, the Federal Association of Practicing Veterinarians (bpt), the German Veterinary Medical Society (DVG), Vetion.de GmbH, Just4Vet, and through selected clinic networks and veterinary chains via email.

The research project was reviewed and approved by the Doctoral Committee and the data protection officer of the university, both acting as the institutional ethical review board. Data collection was carried out in accordance with the ethical guidelines for research involving human participants and in compliance with the university’s internal data protection regulations. All data were stored in pseudonymized form and used exclusively for scientific analysis. The complete questionnaire is provided in Supplementary File 1.

### 2.7 Data analysis

After data collection was completed, all datasets were cleaned and checked for accuracy, plausibility and completeness. Incomplete responses were excluded from the analysis. The data were exported from LimeSurvey, captured, and analyzed using Microsoft Excel for Mac 2024 (Microsoft Corp., Redmond, WA, USA).

Descriptive statistics, including frequency distributions and percentages, were used to summarize the findings and to explore associations between categorical variables. A ranking scale was applied to questions in which respondents indicated which activities were most likely to have led to errors and which factors contributed to these errors. Twelve possible answers were ranked from 1 to 12. Participants were not required to rank all items (activities). To summarize responses across incomplete rankings, a weighted ranking approach was applied. Responses were assigned points based on their rank position, with 12 points allocated to the highest-ranked item and decreasing incrementally to 1 point for the lowest-ranked item. The results were then analyzed based on total weighted scores, allowing identification of the activities and factors that were relatively prioritized by respondents as being associated with errors.

Free-text responses were grouped and analyzed according to overarching themes. The analysis was conducted manually by the authors using a reflexive thematic analysis by Braun and Clarke, a widely used approach for identifying, analyzing, and organizing patterns within qualitative data (17, 18). In this approach, analysis goes beyond simple description: themes are developed through an interpretative process to capture shared meanings across participants’ responses, rather than merely sorting statements into categories. The free-text responses were analyzed by the first author, a German, female, practicing veterinarian, whose professional background provided an insider perspective that informed the interpretation of the data. To enhance reflexivity and analytic rigor, the emerging themes were subsequently reviewed and discussed with the co-authors, who contributed complementary outsider perspectives based on their backgrounds in veterinary education and psychology. The responses were translated into English for the purpose of this publication.

## 3 Results

### 3.1 Demographic and personal data

The analysis encompassed a total of 1793 replies, of which 1,102 questionnaires were completed and formed the basis for the analysis. Among all participants, the majority (82%) were women. More than half of respondents had obtained their veterinary licensure more than 15 years ago (53%). The median age was 44 years, with ages ranging from 24 to 77 years. Further demographic details of the respondents are presented in Table 1. Most participants reported treating between 11 and 20 animals per day (38%). Most participants (75%) worked in a veterinary practice, and 58% indicated that they were employed. Further details on the distribution of respondents regarding their veterinary position and workplace are presented in Table 2.

**Table 1:** Distribution of respondents according to years since veterinary licensure, gender, and age distribution.

**Answers**	**Percentage**	**Responses**
Years since veterinary licensure		
<5 years	16%	*n* = 175
6–10 years	16%	*n* = 176
11–15 years	15%	*n* = 168
>15 years	53%	*n* = 583
Gender distribution		
Women	82%	*n* = 904
Men	17%	*n* = 189
Diverse/Non-binary	<1%	*n* = 1
No answer	<1%	*n* = 8
Age distribution		
20–30 years	11%	*n* = 126
31–40 years	27%	*n* = 300
41–50 years	26%	*n* = 290
31–40 years	27%	*n* = 247
>67 years	2%	*n* = 27
No answer	<1%	*n* = 8

Percentages refer to all respondents with complete questionnaires (*N* = 1,102, 100% of all participants).

**Table 2:** Distribution of responses regarding the average number of patients treated per veterinarian per day, professional position, type of workplace, and employment type.

**Answers**	**Percentage**	**Responses**
Average number of patients treated per veterinarian per day		
<10 Patients	20%	*n* = 220
11–20 Patients	38%	*n* = 422
21–30 Patients	21%	*n* = 231
>31 Patients	17%	*n* = 186
Unknown	4%	*n* = 43
Professional position		
Intern	1%	*n* = 15
Doctoral Candidates	2%	*n* = 19
Veterinarians in Training (refers to veterinarians undergoing specialist training (e.g., residencies))	10%	*n* = 109
General Practitioners	72%	*n* = 796
Specialist Veterinarians	15%	*n* = 169
Senior Veterinarians (refers to senior clinicians or supervising veterinarians (comparable to senior consultants))	14%	*n* = 155
Other	6%	*n* = 61
Type of workplace		
Practice (primary care facilities without 24-h emergency services)	75%	*n* = 825
Clinic (facilities providing 24-h emergency care)	17%	*n* = 183
Large Veterinary Employers (e.g., AniCura/Evidensia)	10%	*n* = 105
Academic Institution (e.g., veterinary schools or university hospitals)	5%	*n* = 56
Other	5%	*n* = 54
Employment type		
Employee	58%	*n* = 634
Partner (co-owners or shareholders)	37%	*n* = 407
Owner	6%	*n* = 61

Percentages are based on all complete questionnaires (*N* = 1,102, 100% of all participants).

### 3.2 Self-reported errors

**Figure 1:**
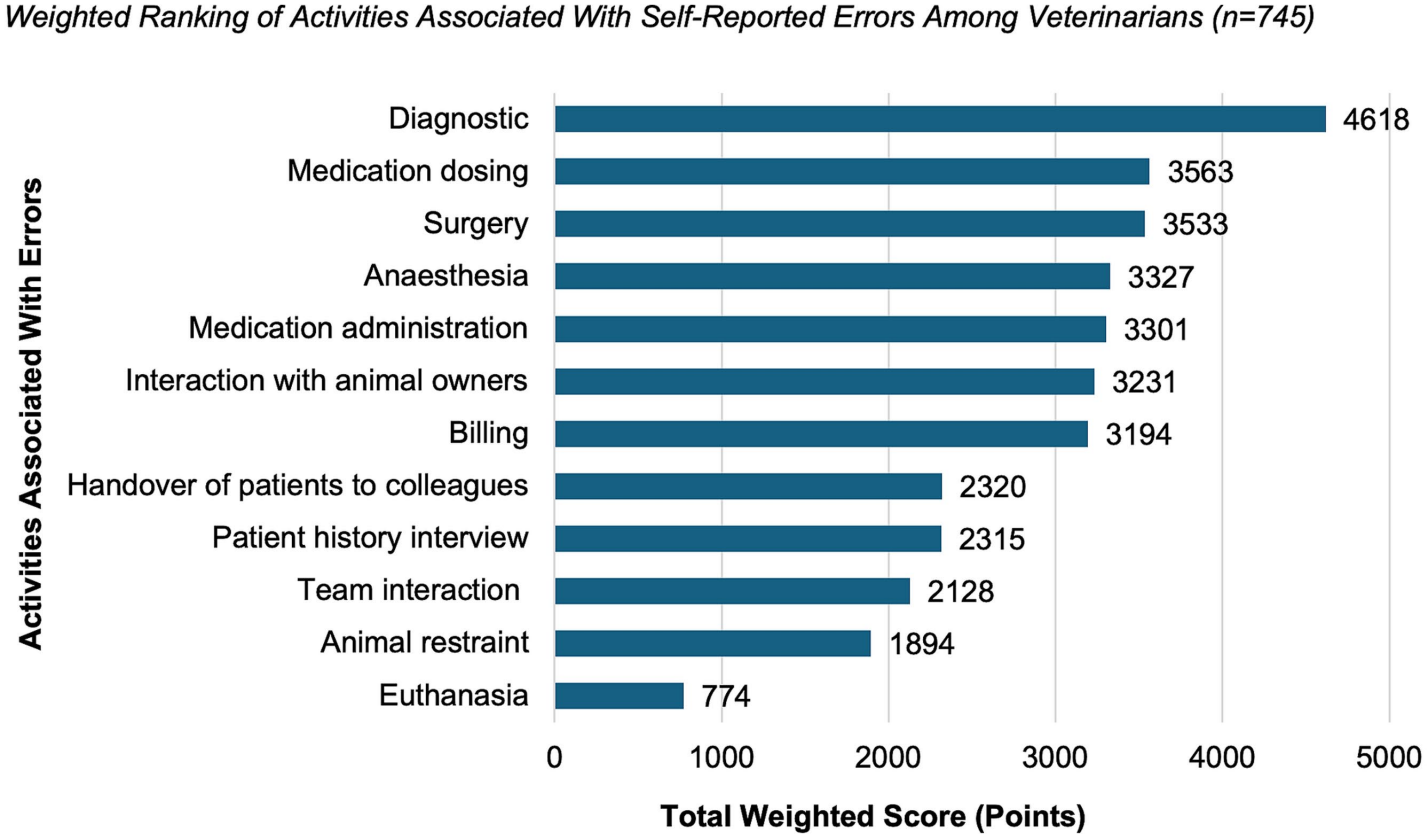
Weighted ranking of activities associated with self-reported errors among veterinarians who reported at least one error (*n* = 745, 68% of all participants). Respondents could select and rank between one and twelve activities. Values represent total weighted scores reflecting the relative prioritization of activities across respondents.

A total of 78% (858/1102) of participating veterinarians had experienced at least one incident in which an animal under their care suffered permanent harm or died. In addition, 68% (745/1102) of all participants reported having disclosed at least one of their own errors that had affected the health or welfare of an animal. This latter group was further analyzed according to years since graduation: 62% (108/175) of veterinarians with less than 5 years of professional experience, 65% (115/176) with 5–10 years, 74% (125/168) with 11–15 years, and 69% (397/583) with more than 15 years had reported at least one such error. Among all participating veterinarians (*N* = 1,102), only those who had previously reported an error (*n* = 745; 68% of all participants) answered the ranking question on error types. Based on the weighted ranking, diagnosis was prioritized most strongly as an activity with errors, achieving the highest overall weighted score (4,618 points). Medication dosing (3,563 points) and surgical procedures (3,533 points) followed with the second-and third-highest weighted scores, respectively (Figure 1).

**Figure 2:**
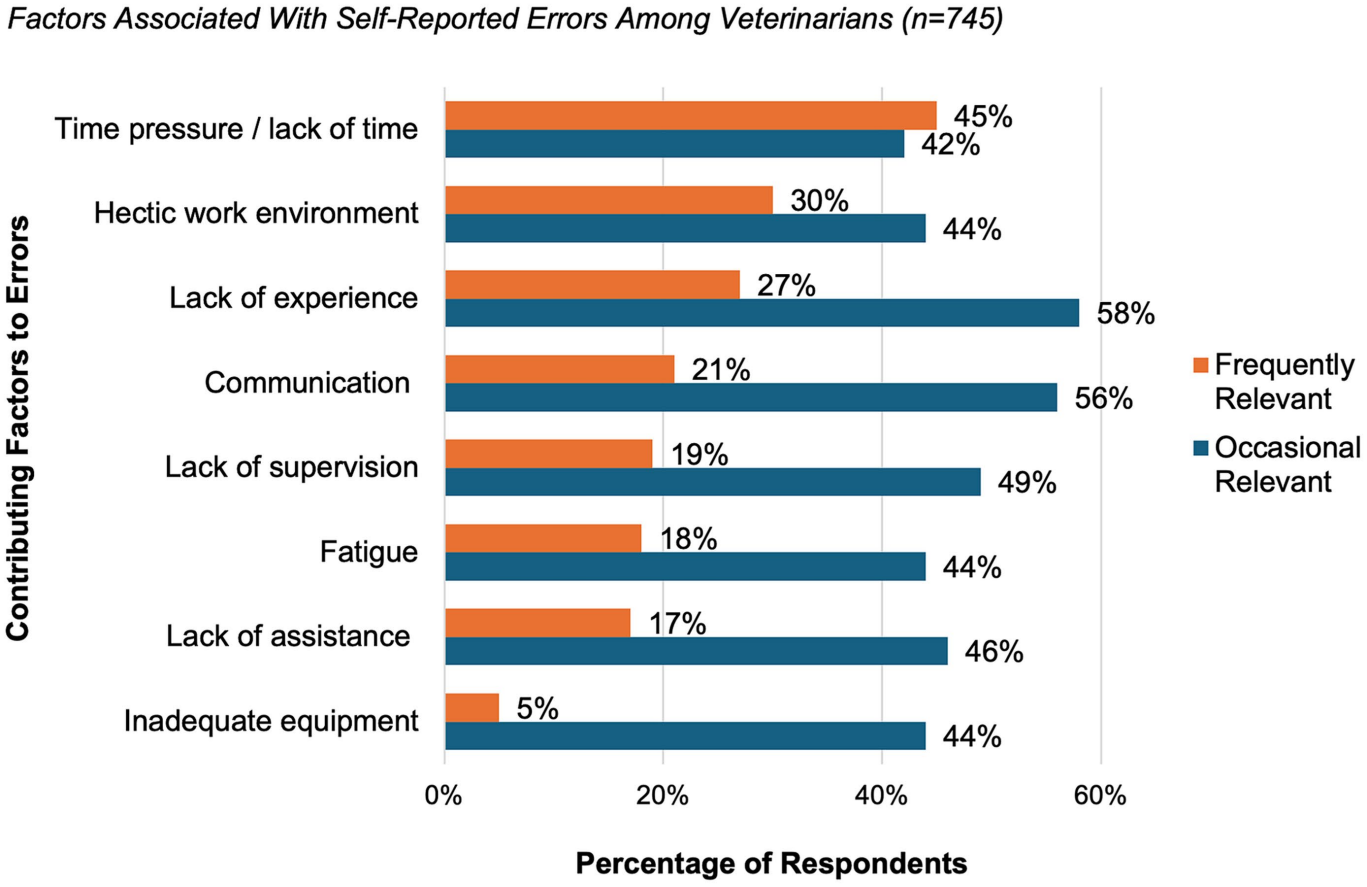
Factors associated with self-reported errors among veterinarians who reported at least one error (*n* = 745, 68% of all participants). Factors were rated individually using a multiple-choice question and categorized as frequently relevant (orange) or occasionally relevant (blue). Results are presented as percentages of respondents. For the category “lack of assistance”, examples include situations such as the absence of veterinary support staff.

When asked about contributing factors to their own reported errors, veterinarians most frequently mentioned time pressure or lack of time (45%), a hectic work environment (30%) and lack of experience (27%). Lack of experience (58%), communication problems (56%) and lack of supervision (49%) were most often rated as occasionally relevant (Figure 2).

Among all participants (*N* = 1,102), 246 veterinarians (22%) stated that they had never reported an error. The most frequently cited reason for not reporting an error was that the errors were considered irrelevant (*n* = 103, 53%). A total of 46 respondents (19%) indicated that no errors had occurred, and another 46 (19%) reported fearing the emotional reaction of animal owners. Concerns about professional consequences were mentioned by 28 respondents (11%), while legal consequences and concerns about personal reputation were each cited by 26 respondents (11%). A total of 24 respondents (10%) also reported concerns about the emotional reaction of supervisors.

### 3.3 Received error reports as supervisors

**Figure 3:**
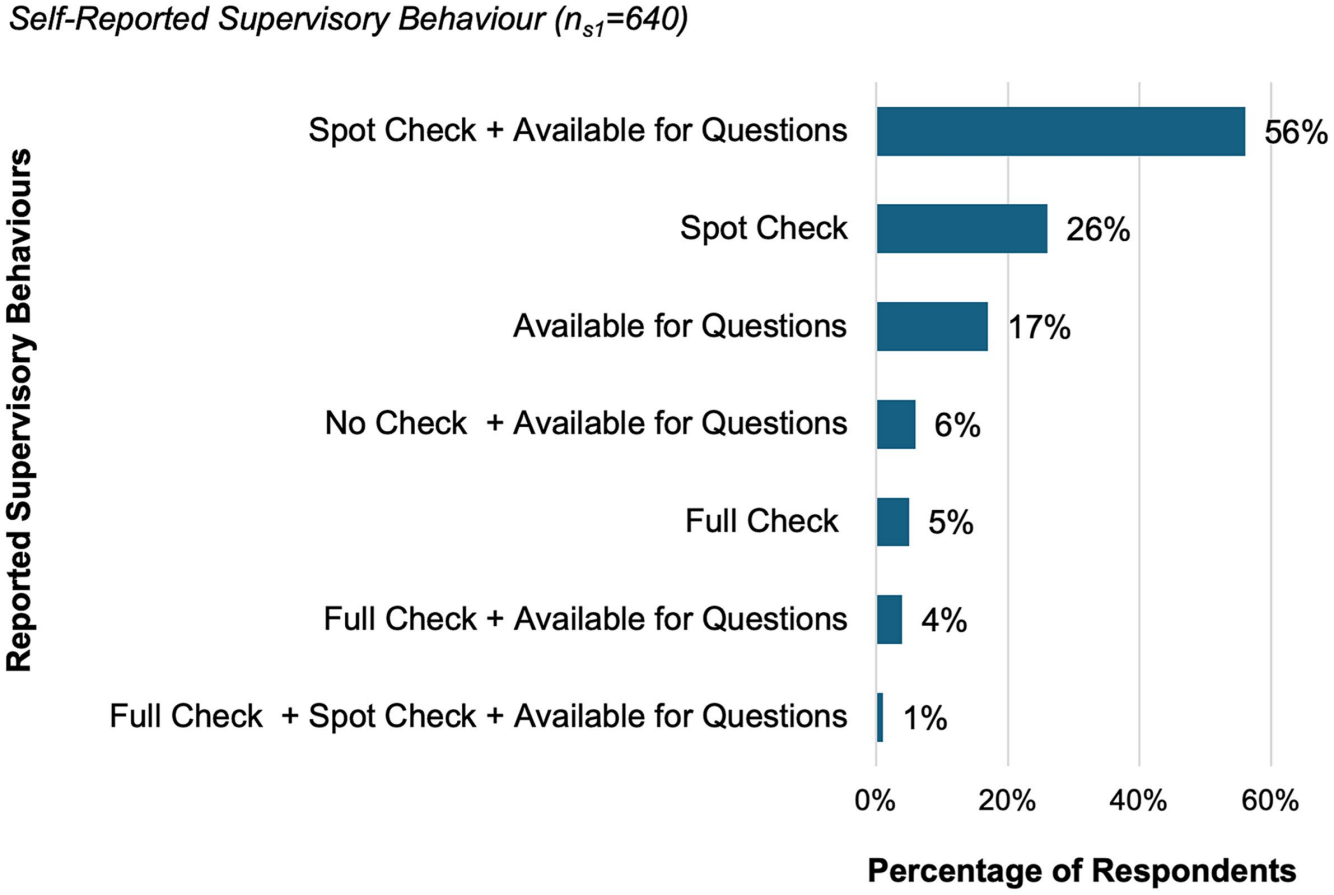
Self-reported supervisory behavior of veterinarians with a supervisory role (*n*_*s*1_ = 640, 58% of all participants). This figure is based on a multiple-choice question in which supervisors indicated how they typically conduct supervision. Spot check refers to random checks of selected tasks, full check refers to a complete review of tasks, and available for questions indicates being accessible for consultation without actively checking. Categories represent either individual behaviors or combinations thereof. Responses accounting for less than 1% were not displayed in this figure.

Among all participants (*N* = 1,102), 58% (*n*_s1_ = 640) reported holding a supervisory role. A total of 45% (500/640) of supervisors reported being responsible for up to ten employees. About 56% of supervisors (301/640) stated that they usually monitor their team members’ clinical activities through random, informal checks while being available for questions. Their self-reported supervisory behavior is shown in Figure 3.

**Figure 4:**
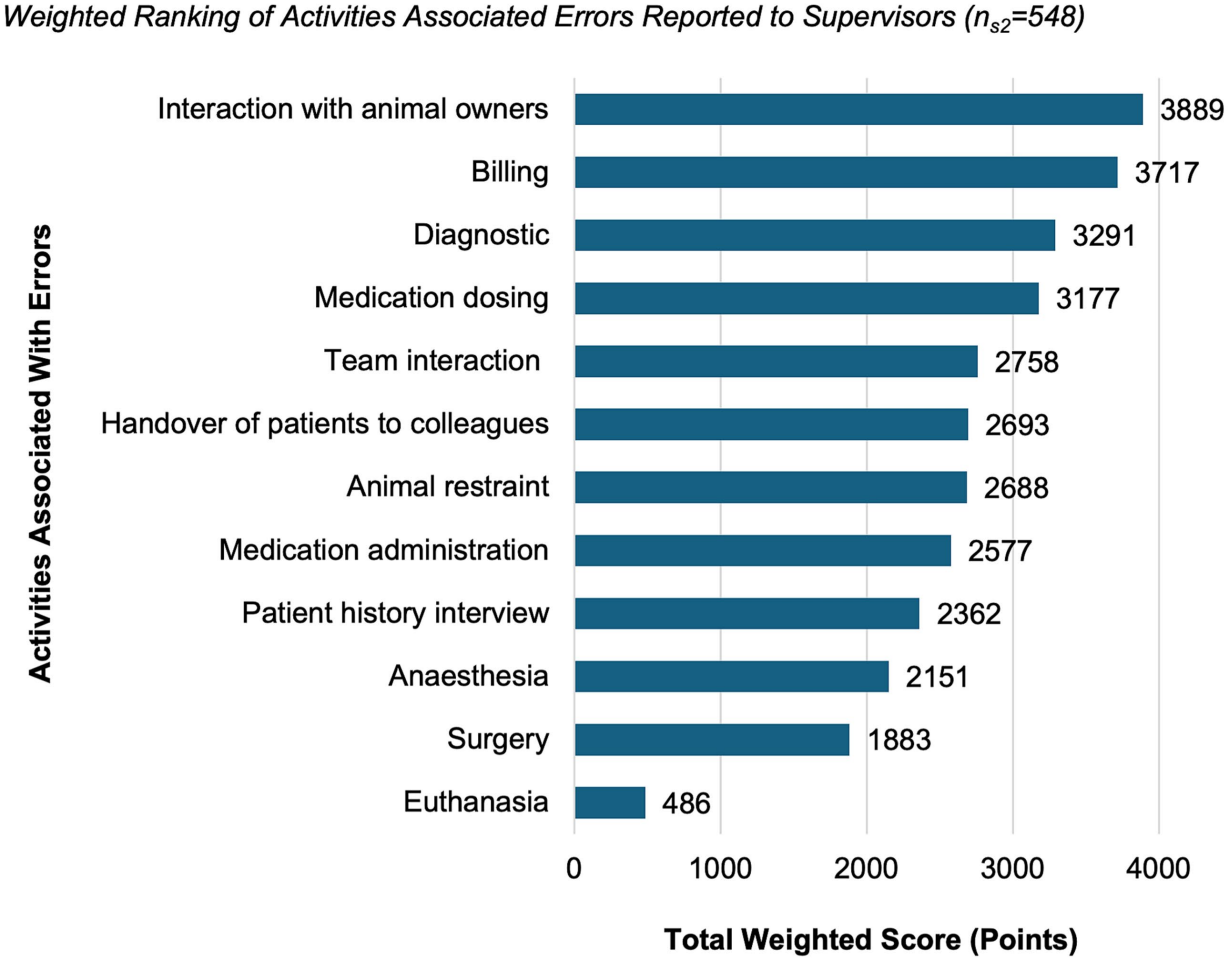
Weighted ranking of activities associated with error reports received by supervisors (*n*_*n*2_ = 548, 86% of all supervisors). Supervisors ranked activities according to their perceived association with error reports they had received. Values represent total weighted scores reflecting the relative prioritization of activities across respondents.

Of the 640 supervisors, 86% (*n*_s2_ = 548) reported having received at least one report of an error that had affected the health or welfare of an animal. Based on the weighted ranking, activities most strongly prioritized in relation to error reports received by supervisors were interaction with animal owners (3,889 points), billing (3,717 points) and diagnosis (3,291 points) (Figure 4).

**Figure 5:**
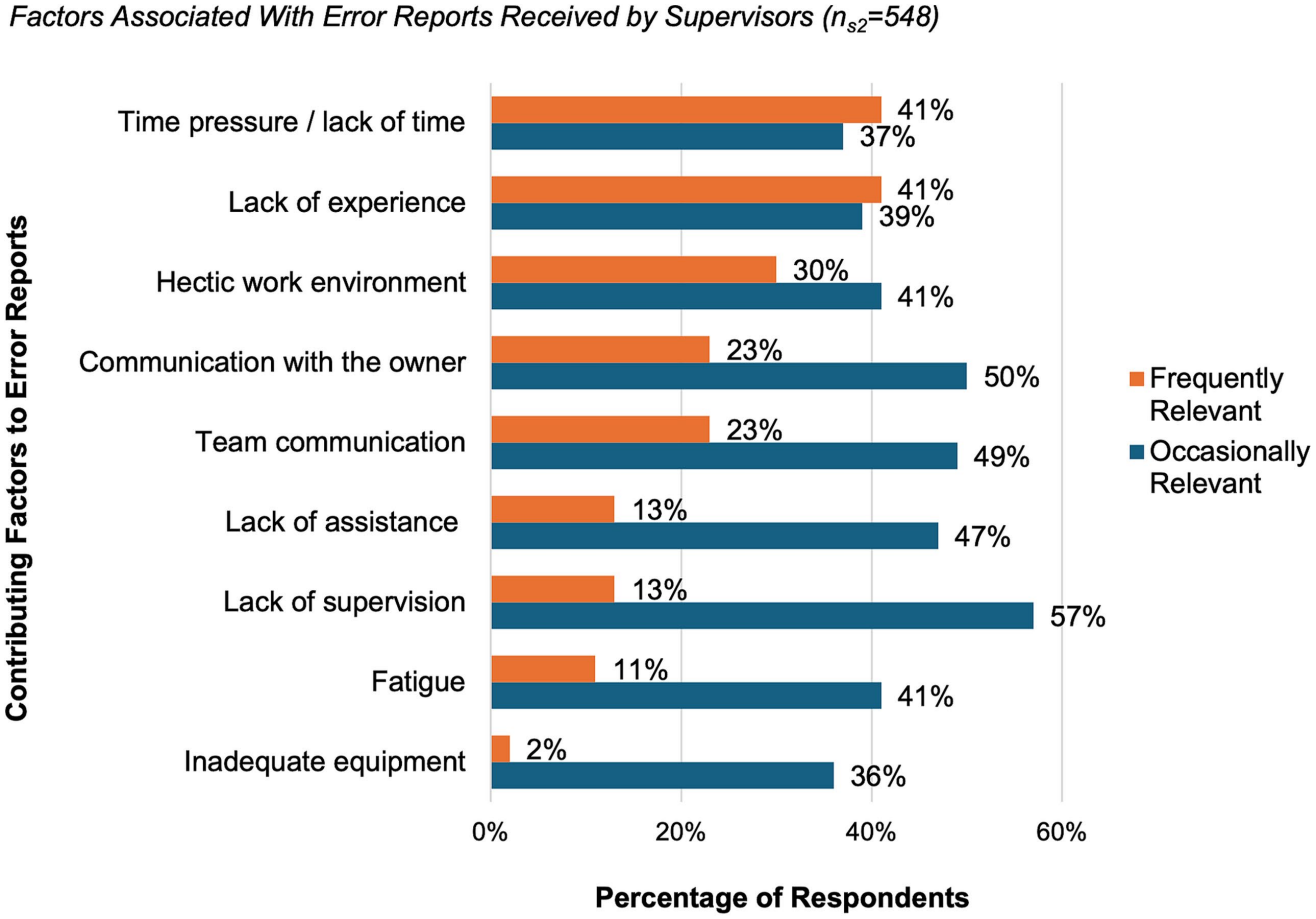
Factors associated with error reports received by supervisors (*n* = 548, 86% of all supervisors). Supervisors rated each factor individually using a multiple-choice question and categorized them as frequently (orange) or occasionally (blue) relevant. Results are presented as percentages of respondents. For the category “lack of assistance”, examples include situations such as the absence of veterinary support staff.

When evaluating factors contributing to errors, supervisors most frequently listed time pressure or lack of time (41%), lack of experience (41%) and a hectic work environment (30%). Factors that were described as contributing occasionally, included lack of supervision (57%), communication problems with animal owners (50%), and within the team (49%). Further frequent and occasional contributing factors reported by supervisors are summarized in Figure 5.

Among the 640 supervisors 7% (45/640) reported that they had never received any error notifications. The most frequent reason given for not having received any error notification so far was that errors had occurred but were considered irrelevant (67%, 30/45). Another common reason was that no errors had occurred (22% 10/45). Other factors included concerns about potential professional consequences (18%, 8/45) worries about negative reactions to error reports, such as rejection or anger (16%, 7/45), concerns regarding personal reputation (13% 6/45), and possible legal consequences (9%, 4/45).

### 3.4 Observed error reports

**Figure 6:**
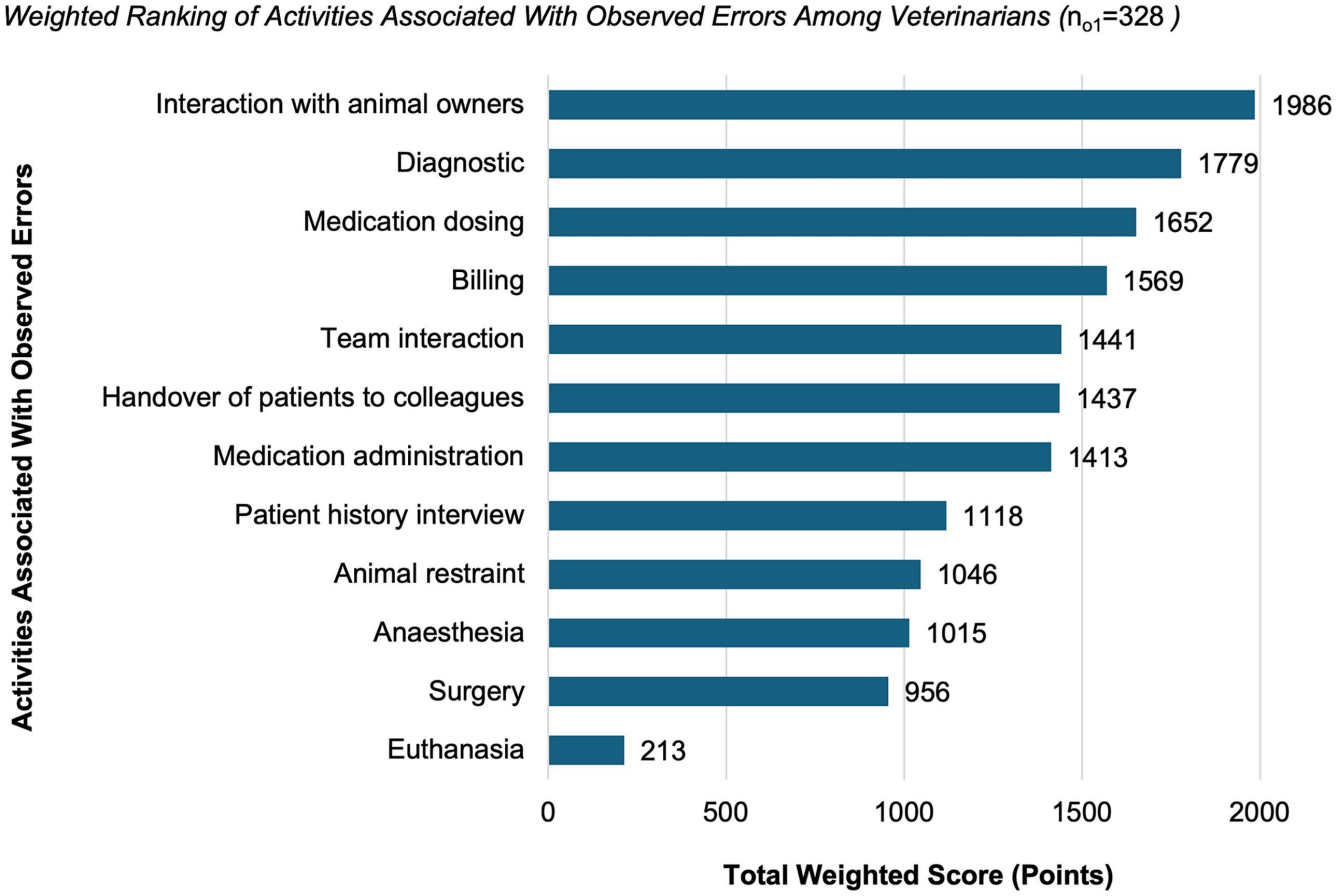
Weighted ranking of activities associated with observed errors among veterinarians (*n* = 328, 30% of all participants). Respondents ranked between one and twelve activities based on errors they had observed in others. Values represent total weighted scores reflecting the relative prioritization of activities across respondents.

Of all participants (*N* = 1,102), 30% (*n*_o1_ = 328) reported having observed at least one error notification that had affected the health or welfare of an animal. The activities most frequently associated with observed errors included interaction with animal owners (1986 points), diagnosis (1779 points), and medication dosing (1,652 points). Further ranked factors are presented in Figure 6.

**Figure 7:**
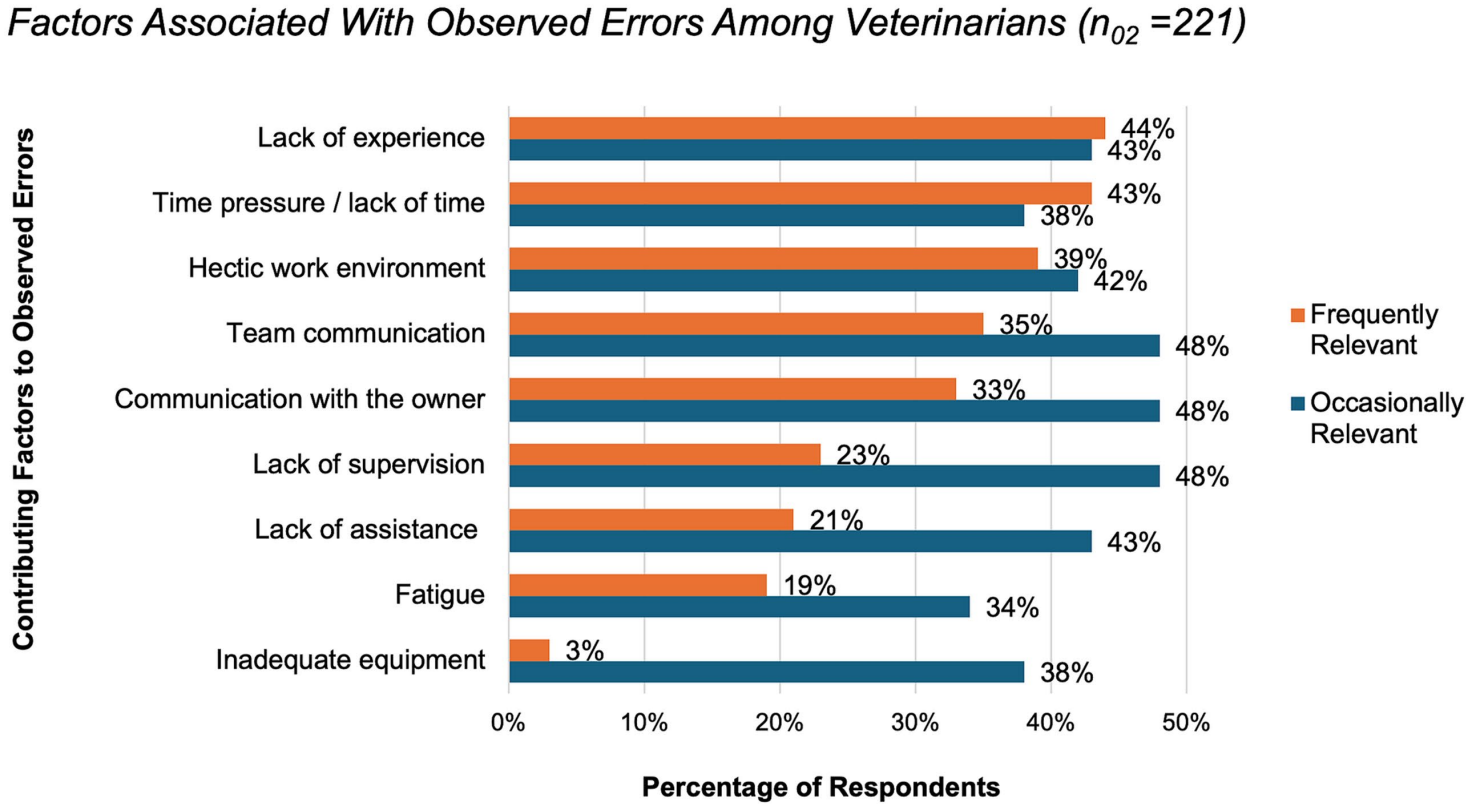
Factors associated with observed errors among veterinarians (*n* = 221, 67% of all participants). Participants rated each factor individually using a multiple-choice question and categorized them as frequently (orange) or occasionally (blue) relevant. Results are presented as percentages of respondents. For the category “lack of assistance”, examples include situations such as the absence of veterinary support staff.

Among the 328 veterinarians who had observed error reports, 67% participants (*n*_o2,_ =221) indicated that they were aware of the contributing factors. The most frequently reported factors were lack of experience (44%), time pressure or lack of time (43%), and a hectic work environment (39%). Communication problems with owners and within the team (both 48%), as well as lack of supervision (48%), were most often rated as occasionally relevant (Figure 7).

### 3.5 Comparison of error perceptions

**Figure 8:**
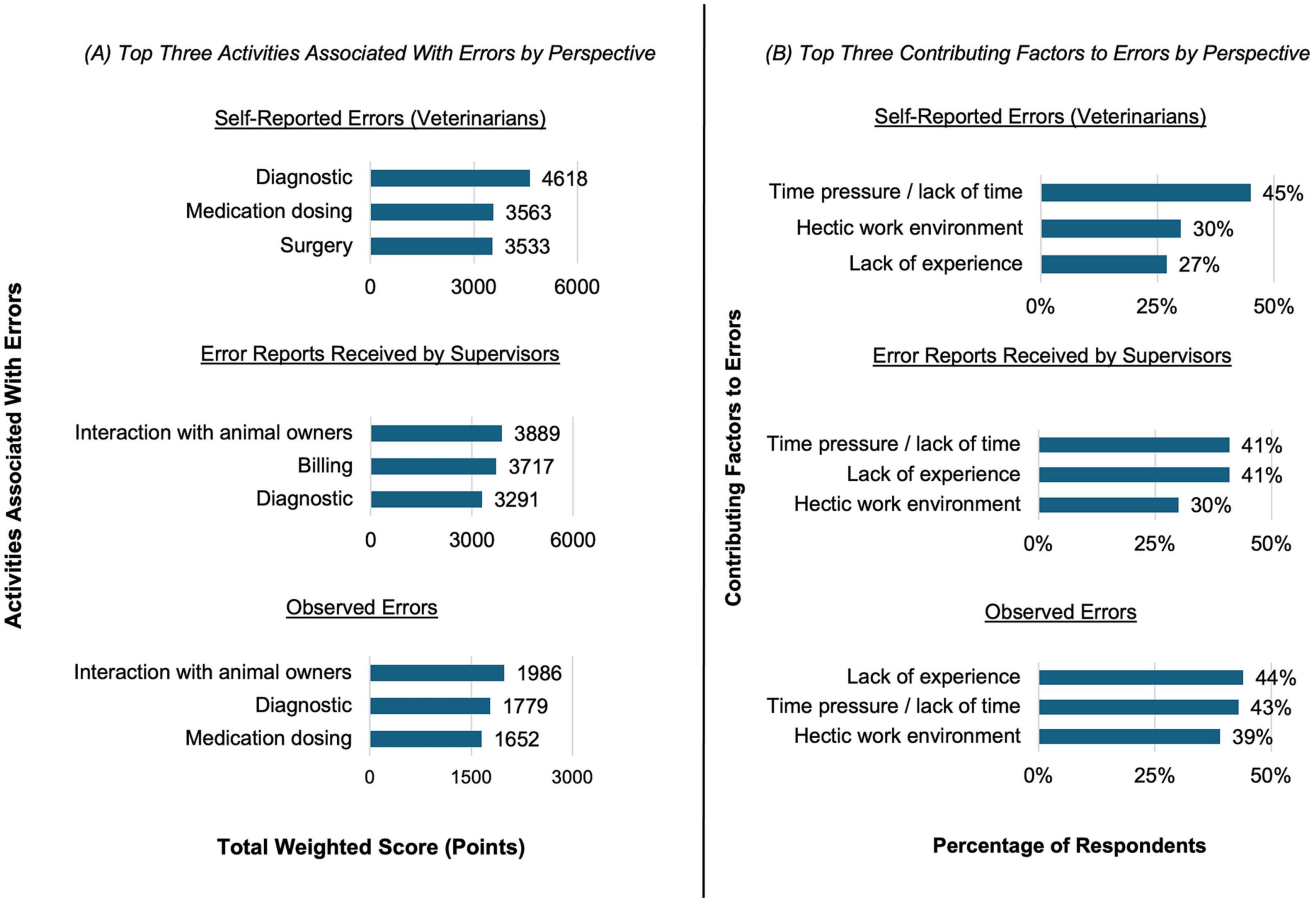
**(A)** Top three activities most strongly prioritized as being associated with errors, based on weighted rankings derived from self-reported errors (*n* = 745, 68% of all participants), error reports received by supervisors (*n*_s2_ = 548, 86% of all supervisors), and observed errors (*n*_o1_ = 328, 30% of all participants). **(B)** Top three contributing factors most frequently reported as being associated with errors, based on self-reported errors (*n* = 745, 68% of all participants), error reports received by supervisors (*n*_s2_ = 548, 86% of all supervisors), and observed errors (*n*_o2_ = 221, 67% of participants who observed errors).

A comparison of the top three perceived error areas shows that, among self-reported errors reported by all participants (*n* = 745), the most frequently mentioned were related to diagnosis, medication dosing and surgical procedure. For errors received by supervisors (*n*_s2_ = 548), the top three areas concerned interaction with animal owners, billing, and diagnosis. In the case of errors observed by all participants (*n*_o1_ = 328), interaction with animal owners, diagnosis, and medication dosing were mentioned most frequently, as shown in Figure 8A.

A comparison of the top three perceived contributing factors shows that, among errors reported by all participants (*n* = 745), the most frequently mentioned factors were time pressure, a hectic work environment and lack of experience. For errors received by supervisors (*n*_s2_ = 548) and errors observed by all participants (*n*_o2_ = 221), the same top three contributing factors were mentioned, albeit in a different order, as shown in Figure 8B.

### 3.6 Error discussion and reporting systems

A total of 54% of all participants (597/1102) reported that no formal error reporting system was available at their workplace. Instead, errors were mainly addressed in informal ways, most commonly through individual conversations (75%). Of all participants (33%) reported having a direct contact person for reporting, while an anonymous reporting system was available in only 4% of cases.

Regular team meetings were used by 36% of all participants (N = 1,102), while special meetings such as Morbidity and Mortality Rounds were less common (19%). Only 5% of all participants stated that a person responsible for quality management was involved in the process, and, in contrast, 6% reported that errors were not followed up at all. A total of 87% of all participants reported that they usually discussed their own errors with colleagues. Just over half of all participants (55%) confided in individuals not directly involved, such as friends, family members, or professional counsellors.

Over half of all participants (54%) reported that, in their workplace, both individual and system-related factors were considered when evaluating errors, while around one quarter (23%) indicated that only the individual was held responsible. Further details can be found in Table 3.

**Table 3:** Availability of error reporting systems in the workplace and perceived causes of reported errors (*N* = 1,102, 100% of all participants).

**Answers**	**Percentage**	**Responses**
Error reporting system at respondents’ workplace		
No error reporting system	54%	*n* = 597
Designated contact person	33%	*n* = 365
Anonymous error reporting system	4%	*n* = 40
Unknown	5%	*n* = 56
Other	4%	*n* = 44
Perception of how errors are typically assessed in respondents’ workplace		
Systemic and individual error	54%	*n* = 595
Systemic error (‘the system is at fault’)	2%	*n* = 26
Individual error (‘the person is at fault’)	23%	*n* = 250
Cause of error not clearly identified	9%	*n* = 103
Unknown	8%	*n* = 87
No answer	4%	*n* = 44

**Figure 9:**
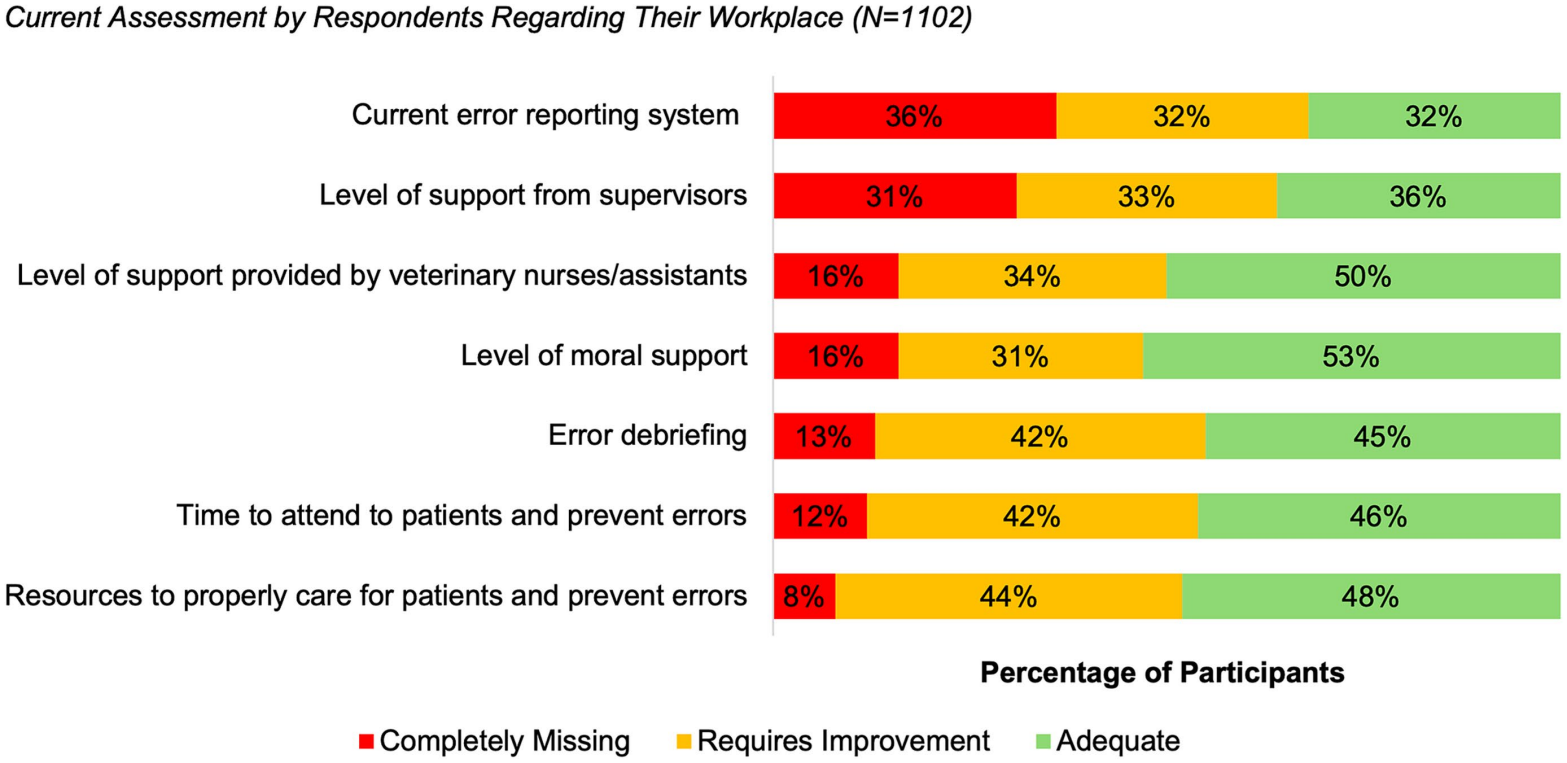
Current assessment by respondents regarding their workplace, categorized as completely missing, requires improvement, or adequate (*N* = 1,102, 100% of all participants).

Figure 9 summarizes an assessment of current practices in error management and prevention by all participants (*N* = 1,102). The currently available error reporting system was rated by participants as either “completely missing” (36%) or “requiring improvement” (32%). Similarly, the level of support from supervisors was rated as “completely missing” (31%) or “requiring improvement” (33%).

### 3.7 Qualitative analysis of free-text responses

Of a total sample (*N* = 1,102), 201 participants (18%) provided free-text responses to the open-ended closing question (“Is there anything else you would like to share with us on this topic?”). The qualitative analysis resulted in a set of interrelated themes. The themes are not mutually exclusive; rather, they reflect overlapping dimensions of a complex professional reality. Overall, the responses were categorized into six main themes: Error Culture and Handling of Errors, Psychological Burden, Positive Examples or Constructive Approaches, The Role of Supervisors, Work Environment and Education, Training and Systems. Below, the themes are presented with illustrative examples drawn directly from participants’ responses and briefly related to existing research:

#### 3.7.1 Theme 1: error culture and handling of errors

The analysis indicated that the prevailing perception of the error culture in veterinary medicine was that it is insufficient. The responses reflected patterns in which errors are often avoided, concealed, or associated with blame. Experiences with a “zero-tolerance” approach to errors were described and were interpreted as burdensome. Several vets reported that, in smaller and family-run practices, open discussion about errors was particularly difficult. This was illustrated by statements such as:

- “Basically, mistakes should of course be avoided. However, especially in our profession, it seems better to conceal them or to gloss over them rather than to admit a mistake directly, because the consequences can be far-reaching and the way they are dealt with leaves room for improvement.”
- “A zero-tolerance error culture led to an enormous feeling of pressure for me. I temporarily left the profession.”
- “The practice is run together with the spouse. This constellation often makes it difficult to have open communication about occurring problems between employees on the one hand and the business partner/spouse on the other.”

Lack of time, insufficient communication, and staff shortages were identified in the data as contributing factors to the occurrence of errors. Overall, the handling of errors was understood as being shaped by the structure of the practice and communication style. This interdependence was illustrated by comments such as:

- “The cause of most mistakes is haste and a lack of care in the preparation and performance of tasks (anesthesia, surgery, medication dosing), both caused by staff shortages.”
- “Mistakes mainly occur due to time pressure, which is increasingly rising because there is no new staff available in the practice.”
- “In my experience, at universities, a high number of patients, time pressure and overwork often lead to mistakes […]”

The points made here reflect characteristics of blame-oriented safety cultures, in which fear of consequences inhibits open communication and organizational learning, as described in safety culture research (8, 19).

#### 3.7.2 Theme 2: psychological burden

The psychological dimension of the topic was prominent across the data set. Feelings of guilt, fear, and emotional strain in connection with errors or the possibility of making them were evident across participants’ accounts. The narratives also reflected pressure from animal owners or supervisors, which was experienced as an additional stress factor. Participants described this emotional burden in personal and persistent terms, for example:

- “The fear of making mistakes, of having forgotten something, and the lack of sufficient time on stressful days are, for me, the greatest stressors and affect me even outside of work.”
- “I stopped working in clinical practice because of fear of making mistakes.”

The analysis further highlighted that academic education provides insufficient preparation for psychological strain and dealing with mistakes. Increasing public visibility of errors, for instance through online reviews, was also reflected in the data. This lack of preparation and increasing pressure were illustrated by statements such as:

- “I think one is not prepared for something like this during one’s studies […] often overwhelmed and more mistakes happen. Nevertheless, good work is still expected.”
- “Doctors still have to work perfectly and without errors. No mistakes are forgiven.”
- “One often feels alone with the feelings of guilt that arise when one has made a mistake. The fear of making a mistake accompanies you, because you know that you have to cope with your own feelings of guilt and with being exposed on Google.”

Psychological support, supervision, and structured opportunities for processing emotionally stressful situations emerged as potential supportive resources. Participants suggested concrete support formats, for example:

- “I would like to see supervision groups established in veterinary medicine, as many colleagues suffer from the pressure to always do everything right, especially when mistakes can have fatal outcomes.”
- “Psychological support is missing.”
- “We do not call it supervision, but cope through shared breaks, eating together and chatting […] or, with a lot of humor, sometimes even dark humor, making mistakes in communication with owners more bearable […] or ‘laughing them away’.”

The emotional narratives resemble what has been described in healthcare as the “second victim” phenomenon, highlighting the psychological impact of errors in environments with limited structured support (20, 21).

#### 3.7.3 Theme 3: positive examples or constructive approaches

Alongside these challenges, the analysis also captured accounts of constructive ways of dealing with errors. The data reflected open communication structures, flat hierarchies, and a respectful team culture that enable joint analysis of errors. Errors are constructed as a natural part of everyday professional life and as opportunities for learning. These constructive practices were illustrated by statements such as:

- “All employed veterinarians and veterinary nurses know that they can speak openly with me about mistakes without severe consequences. We are more interested in finding a quick solution.”
- “Open discussion of mistakes is well established in our team; we all learn from it […]”
- “[…] we discuss errors directly or in team meetings without assigning blame […]”

Positive experiences with honesty towards animal owners were also reflected, contributing to trust-building. Openness and transparency on the part of management were understood as supportive factors that foster reflection and professional development. Participants linked transparency to trust-building and learning, for example:

- “[…] We focus on finding solutions quickly rather than assigning blame.”
- “Mistakes happen and everyone should be allowed to admit them without having to fear consequences.”
- “Error management is not about blame. Inherent problems, system errors and lack of knowledge should be identified and eliminated.”

These responses illustrate elements of a learning-oriented and generative safety culture, characterized by transparency and collective reflection (19).

#### 3.7.4 Theme 4: the role of supervisors

The role of supervisors and leaders emerged as a central aspect in how errors were handled. Different reactions to errors are described, some of which are perceived as personal or inconsistent. A lack of recognition and an emphasis on mistakes are mentioned, alongside a wish for clear communication, empathetic leadership, and regular supervision. This contrast was illustrated by statements such as:

- “[…] there is no constructive follow-up discussion, only ‘nonsense’ and ‘complaining’.”
- “[…] employers and leaders also need to actively promote this […]”

A transparent and consistent error culture at the management level was constructed as essential for handling errors constructively. Participants described how leadership behavior directly shaped openness and psychological safety, for example:

- “Old mistakes are constantly brought up in front of others […]”
- “[…] My boss himself never makes mistakes… […]”
- “Reactions to mistakes are very personal in our practice. The same incident is judged differently depending on the team member, and that is extremely unfair.”

The accounts underscore the role of leadership behavior in shaping psychological safety and consistency in error management, consistent with prior findings in veterinary healthcare research (22, 23).

#### 3.7.5 Theme 5: work environment

Structural and organizational conditions were interpreted as influential factors in error management. Economic changes were mentioned as advantages but also as barriers in relation to thorough diagnostics. The differing, and in some cases entirely absent, ways of dealing with this among colleagues and practices were also reflected in the data. These constraints were illustrated by statements such as:

- “High fees prevent necessary examinations for many owners, so that veterinarians often have to treat without thorough diagnostic certainty. This causes mistakes and anesthetic incidents.”
- “Since the new fee schedule, many things have improved, because we work somewhat less intensively and therefore have a bit more time […]”
- “[…] exchange with neighboring practices is impossible, as they are all competitors.”
- “Error discussions […] with referring veterinarians are completely absent or are often classified as ‘nest-fouling’. Criticism is often avoided for economic reasons (as referrals may stop) and/or due to resistance to criticism.”
- “I was always able to ask questions easily and also use a ‘telephone joker’ for error prevention (fresh out of university).”

Structural and economic pressures reflect broader workload pressures described in the veterinary industry (24), highlighting also the common conflict of providing compassionate care while constrained by financial frameworks.

#### 3.7.6 Theme 6: education, training, and systems

Another thematic area concerns education, training, and systems. The university education was widely described as insufficient in preparing students for the practical handling of errors and the emotional demands of the profession. The teaching of communication and self-reflection skills was identified as an area requiring further development. Participants emphasized this need, for example:

- “I think one is not prepared for something like this during one’s studies […] often overwhelmed and more mistakes happen. Nevertheless, good work is still expected.”
- “Leadership and communication with animal owners should already be practiced at university.”
- “Young veterinarians should spend a sufficiently long time working alongside an experienced veterinarian and be taught.”

A need for structured systems for error detection and analysis emerged across the data, as well as for approaches that support adequate management of available resources. Furthermore, the integration of structures for learning and practicing an error culture into everyday professional practice was emphasized as a way to improve the long-term safety and quality of veterinary work. Participants formulated concrete suggestions, for example:

- “The willingness of the institution is there (error reporting system), but it is hardly used or supported.”
- “CRM and CIRS systems like those used in human medicine are long overdue.”
- “[…] should not be a leisure activity, but official working time and part of the job.”

Participants’ calls for structured supervision, communication training and formal reporting systems highlight a perceived gap between formal education and the non-technical competencies required for safe clinical practice, echoing recent discussions on human factors in veterinary medicine (25).

## 4 Discussion

### 4.1 Error prevalence

This study demonstrates that errors in veterinary practice are not an exception but a regularly occurring phenomenon. In total, 78% of participants reported having experienced at least one case in which an animal under their care was permanently harmed or died, and 68% stated that they had disclosed at least one of their own errors. Notably, 62% of survey participants with less than 5 years of professional experience had already disclosed an error, indicating that in this sample, serious incidents occur within the first years of professional practice. Errors are therefore part of clinical reality, a finding that has also been well documented in human medicine (16, 26) and is increasingly recognized within veterinary healthcare (6, 27). Beyond patient safety, these conditions also affect professionals themselves. The “second victim” phenomenon highlights the emotional burden experienced by veterinarians following errors, which is exacerbated by lack of support and punitive cultures (28). The presence of errors calls for an “informed culture” that actively collects and analyses risk- and incident-related information, rather than suppressing it (8). A structured, learning-oriented error culture is therefore essential both for risk reduction and staff wellbeing.

### 4.2 Perspectives and perception

Perceptions and evaluations of errors are strongly dependent on professional role. Previous studies, particularly Oxtoby et al. (29) and Kinnison et al. (22), showed that errors in veterinary practice occur primarily in diagnostics, medication, and communication. Our participants perceptions confirm this pattern and additionally demonstrate clear role-specific differences in how errors are recognized and interpreted.

Self-reported errors most commonly involved diagnostic mistakes, medication-related issues or surgical procedures, whereas supervisors more frequently received reports concerning communication with owners, billing, or diagnostic procedures. Observing colleagues primarily noticed client-related interactions and visible clinical or dosing errors. These differences illustrate how professional responsibilities shape what is perceived as an error: clinicians mainly identify technical or clinical mistakes arising from their own decisions; supervisors encounter externally visible errors that surface through complaints or administrative discrepancies; and team members observe what appears within their immediate work environment.

Because 58% of respondents held supervisory roles and 86% of them had received at least one error report, most often in relation to incidents that were visible or had already escalated, the findings suggest reported errors tend to involve more apparent cases. Externally noticeable errors are more likely to reach leadership, while internal clinical or procedural mistakes often remain unreported. Thus, each perspective captures only part of the overall error landscape, and leadership significantly influences which errors become discussable at all. This pattern can be interpreted through Westrum’s typology: in less mature (pathological or bureaucratic) cultures, information tends to be filtered vertically, and “bad news” is either punished or managed defensively, whereas generative cultures promote active information sharing and collective learning. The role-dependent visibility of errors observed here therefore points to systematic ‘blind spots’ created by hierarchical and organizational information flows, not merely by individual reluctance (8, 19, 30). Similar patterns of selective perception are well described in human medicine, where communication failures, often rooted in unclear responsibilities or hierarchical barriers, are among the most common contributors to clinical incidents (31–34). Fear of legal consequences, loss of reputation or loss of trust is regarded as one of the strongest inhibiting factors for open error communication (35). At the same time, studies show that patients explicitly prefer openness and transparency about errors. A recent study among animal owners indicates that the majority wish for honest disclosure, taking responsibility and even the introduction of anonymous reporting systems (36).

The concept of ‘Just Culture’, developed by James Reason (8), offers a theoretical framework for understanding these dynamics. In complex systems, errors arise from the interaction of individual and organizational weaknesses, as illustrated in Reason’s well-known “Swiss Cheese Model.” Importantly, a Just Culture is not equivalent to a “No-Blame Culture.” While individual errors are understood as manifestations of systemic weaknesses, accountability remains a central element, particularly in cases of deliberate deviation from standard operating procedures or when such deviations cannot be adequately justified. The core principle of a Just Culture therefore lies in a fair, consistent and predictable response to errors that enables learning and improvement, without resorting to punitive or purely blame-oriented practices (37). Uncertainty about organizational reactions, fear of blame, and selective reporting indicate that responses to errors may be perceived as inconsistent or unpredictable, conditions that directly undermine both reporting behavior and learning (8, 37).

### 4.3 Contributing factors

Across all perspectives, time pressure, a hectic working environment, and lack of experience were identified as the most frequent contributing factors. Communication problems and insufficient supervision were also commonly reported, corroborating earlier findings by Kinnison et al. (22).

These results indicate that errors are primarily perceived as structurally driven rather than the result of individual misconduct. Non-technical skills such as communication, decision-making, and situational awareness remain insufficiently embedded in veterinary education. Moreover, a look at veterinary education shows that expectations and reality diverge: The European Association of Establishments for Veterinary Education (EAEVE) defines accreditation standards and learning objectives for veterinary curricula. These include so-called “Day One Competences” (D1Cs), which describe the skills and behaviors expected of graduates at the start of professional practice. For example, D1C 1.15 explicitly requires that veterinarians “regularly engage in self-audits and peer-review processes in order to improve their performance” (38). Evidence from models such as Kolb’s experiential learning and analyses of VetSafe (39, 40) suggests that many contributing factors, especially those related to communication, supervision, training, and work organization, are modifiable.

### 4.4 Barriers to discussing and reporting errors

Barriers to error communication are reflected in the fact that many participants never disclosed their own errors (22%). The reasons given for not discussing errors included a lack of perceived relevance, fear of negative reactions from animal owners, concern about professional or legal consequences, and discomfort within the team.

Supervisors also reported that error notifications were sometimes absent, partly because they assumed that no errors had occurred, and partly because there appeared to be reluctance within teams to raise errors. These patterns mirror findings from human medicine and aviation, where hierarchies, role expectations, and fear of sanctions are described as key causes of underreporting (41–43). For example, the Safety Culture Ladder, developed by Hudson, describes how organizations differ in their approach to safety and errors across successive levels of maturity, ranging from cultures in which errors are hidden or addressed only reactively, to more advanced cultures where risks are openly discussed and systematically used for learning. Interpreted within this framework, the barriers identified in the present study are more consistent with early or intermediate stages, in which error reporting tends to be defensive or episodic rather than a routine part of everyday work (44). Similar dynamics have also been reported in veterinary settings, where interprofessional boundaries, unclear role expectations, and power asymmetries within teams limit open communication about errors (45).

### 4.5 Debriefing practices and formal error reporting systems

The results of this study show that structured reflection processes and formal error reporting systems are, to date, insufficiently established in veterinary practice. More than half of the respondents (54%) stated that no formal error reporting system exists in their institution, and only 4% reported the possibility of anonymous reporting. Consequently, error management remains predominantly informal and only marginally embedded in organizational learning. At the same time, established initiatives demonstrate feasibility within veterinary medicine. Systems, such as the AniCura Patient Safety Improvement System (APSIS) and VetSafe, illustrate that standardized, non-punitive reporting pathways can support the identification of safety-relevant events and the derivation of improvement measures (46). APSIS exemplifies organization-internal reporting structures, while VetSafe serves here as an example of a formalized system that promotes transparency and systemic responsibility, allowing both anonymous and named reporting (40).

Despite this evidence, error management in many institutions continues to take place predominantly in an informal manner: 75% of respondents reported that errors are primarily addressed in one-to-one conversations. While such discussions may provide short-term emotional relief, they substantially limit collective reflection as well as systematic analysis of contributing factors. This pattern corresponds to the phenomenon known from human medicine as the “invisible burden of error”, in which events are processed on an individual level but are rarely documented or translated into organizational learning processes (47). In Reason’s model, the dominance of informal one-to-one conversations in the veterinary context examined in this study suggests that the ‘reporting’ and ‘learning’ components of an informed culture are currently underdeveloped, meaning that information is not reliably transformed into shared organizational knowledge (8).

The limited use of structured formats such as morbidity and mortality conferences or Significant Event Audits (SEA) is further reflected in the fact that only 36% of respondents reported regular team meetings and just 19% reported *ad hoc* team meetings, despite these formats being described in the literature as key elements for transparency, non-blaming exchange, and organizational learning (48, 49).

In addition, the results show that engagement with errors is often shifted beyond the institutional setting: while 87% of respondents reported discussing errors with colleagues, more than half (55%) sought conversations with external individuals such as friends or family members. This externalization can be interpreted as an indication of limited psychological safety and a lack of trust in internal structures, hallmarks of an immature error culture (30, 50). Importantly, externalization should be understood less as an individual coping preference and more as an organizational symptom: when internal structures are perceived as unsafe, informal and private spaces become the default arena for processing errors (8, 30). Psychological safety in this context is less an individual attribute than the result of reliable structural conditions, in particular predictable organizational responses, consistent leadership behavior, and clearly defined responsibilities (30, 37).

At the same time, successful examples from veterinary practice, such as in small animal medicine (51), residency programs (52), or equine anesthesia (53), demonstrate that structured debriefing formats are also feasible in the veterinary context and can contribute to fostering an open, learning-oriented approach to errors. These findings underline that formal reporting systems and structured reflection processes are particularly effective when embedded within a coherent organizational framework grounded in transparency, non-punitiveness, and systemic accountability.

### 4.6 Assessment of existing structures and resources

The assessment of existing structures relevant to a functioning error culture revealed substantial deficits across several key domains. Overall, 68% of respondents rated the error reporting system in their institution as either “completely missing” (36%) or “requiring improvement” (32%). This indicates that errors and near misses are frequently not documented in a systematic manner. The literature emphasizes that low-threshold, anonymous and non-punitive reporting systems are essential for organizational learning and for identifying latent systemic risks (54, 55).

Support from supervisors was also evaluated critically: 64% of respondents rated leadership support as insufficient or inconsistent. This aligns with findings by Kinnison et al., Mosedale and Oxtoby & Mossop, who highlight the key role of leadership in building trust, modelling transparency and promoting structured reflection processes (22, 23, 51). This assessment confirms the previously described structural bias in perception and reporting: errors that are visible to leadership levels or have escalated are more frequently addressed, whereas internal clinical or procedural deviations are less likely to enter formal communication and learning processes. Against this background, it becomes evident that limited leadership support influences not only individual reporting behavior but also constrains organizational learning as a whole. Leadership behavior is a key determinant of whether information is rewarded, tolerated, or punished, and therefore shapes the entire reporting climate (19).

Perceptions of moral support further reinforce these findings. A total of 47% of respondents considered emotional support inadequate. Rather than representing a separate issue, this result strengthens earlier observations regarding limited psychological safety: emotional backing is often regarded as a prerequisite for speaking openly about errors, yet many respondents indicated that such support was lacking. Consistent with the patterns described above, insufficient psychological safety contributes to error-related discussions being shifted to private settings rather than taking place within the institution (30, 56).

Structured opportunities for discussion and review were also assessed critically. A total of 55% of respondents reported that formats such as morbidity-and-mortality (M&M) rounds or Significant Event Audits (SEA) were missing or insufficient. This finding mirrors earlier results on the low prevalence of formalized reflection structures. In contrast, established formats such as SEA, Critical Incident Reporting Systems (CIRS) and M&M rounds are widely recognized as essential mechanisms for transparency, non-punitive exchange and collective learning (48, 49).

Finally, more than half of respondents rated both their available time (54%) and their staffing or material resources (52%) as inadequate to meet clinical demands and prevent future errors. These findings are consistent with research by Bartram et al. (24), who identified resource shortages and workload intensity as central stressors in veterinary practice, as well as evidence from human medicine demonstrating that inadequate temporal and organizational resources impair clinicians’ ability to detect, manage and prevent errors (57). In light of demographic developments and the increasing commercialization of the veterinary sector, such resource limitations are unlikely to improve substantially in the near future. Consequently, sustainable strategies will need to prioritize optimizing clinical workflows and supporting staff in working safely under constrained conditions, for example through structured teamwork approaches (e.g., Crew Resource Management) and, where appropriate, selected digital or AI-supported tools. The use of such tools may be guided by ethical principles such as transparency, proportionality, and clear purpose limitation, with a focus on supporting organizational learning through data aggregation and pattern recognition. Interpretation and responsibility for action should continue to lie with human professionals, rather than being delegated to automated systems.

### 4.7 Integrated synthesis and implications

The results of this study demonstrate that errors in veterinary practice occur regularly. The importance, however, lies less in the fact that they occur, but rather in the way structural, organizational and communicative weaknesses influence how errors emerge, are perceived and are handled. The three perspectives examined – self-reported, received and observed errors – reveal that error perception is strongly role-dependent. Clinically active veterinarians mainly identify diagnostic and medication-related errors, managers predominantly perceive externally visible incidents such as communication or billing problems, and staff members primarily register what becomes apparent within their immediate working environment. As a result, only partial segments of the overall error landscape become visible within institutions. Since errors are perceived differently depending on professional role, institutions require structures capable of integrating these perspectives. Interdisciplinary case discussions, regular team meetings and standardized formats for reflection can help reduce “blind spots” and provide a more complete picture of the actual spectrum of errors (30, 58). This integration can be conceptualized as strengthening the ‘informed culture’ by improving information flow through observable organizational artefacts such as reporting systems, debrief formats and routine peer review (8, 19, 59).

Across all three perspectives, the most frequently identified contributing factors – time pressure, workload, lack of experience, communication difficulties and insufficient supervision – point clearly to structural influences rather than individual shortcomings. These factors are, at least in principle, systemically modifiable through leadership, team organization, training, resource allocation and continuing education. At the same time, the findings indicate that many institutions currently lack a learning-oriented error culture: reporting pathways are often unclear or absent, managerial reactions inconsistent, structured reflection formats rare and resources limited. Existing systems such as VetSafe, APSIS and M&M conferences demonstrate that such structures can function successfully when implemented consistently (40, 46, 60).

The free-text responses add qualitative depth to these findings. Some respondents report positive experiences of open communication that foster trust, while many others describe an everyday reality shaped by uncertainty, fear of blame, hierarchical barriers and a shift of error-related discussions into private settings due to a lack of psychologically safe institutional structures. This discrepancy between the theoretical aim of a Just Culture and the lived experience of many veterinarians highlights the importance of reliable and supportive leadership behavior. The results also point to a gap between educational objectives and clinical reality. Self-reflection, peer review and human-factors competencies must be more firmly embedded in undergraduate and postgraduate training. From an educational and systems perspective, this implies a more systematic integration of human-factors principles into veterinary curricula and clinical routines. Effective veterinary care depends not only on clinical expertise but also on structured communication, clear role allocation within teams, the use of cognitive aids such as checklists or algorithms, and routine debriefing after significant clinical events (25). Simulation-based training can further support the development of these competencies in a safe learning environment. Participants’ calls for improved preparation during university training, structured supervision for early-career veterinarians, and the implementation of CRM or CIRS systems closely align with these recommendations. Embedding these elements formally within education and institutional practice would therefore represent a concrete step toward strengthening both patient safety and professional resilience. At the same time, more than half of respondents perceived insufficient time and staffing resources as contributing factors to errors. This underscores the importance of workforce planning, clear prioritization, and structured decision-making support in daily practice.

At the same level, transparent and non-punitive reporting structures should be established (16, 61), ensuring that reported events are systematically analyzed and translated into institutional learning. Leadership is required that provides predictable, supportive and open reactions, communicates personal errors transparently, and establishes clear rules for dealing with errors. To achieve this, organizations need explicit decision rules that distinguish human error, at-risk behavior and reckless behavior, so that accountability is perceived as fair and predictable rather than arbitrary (8, 37). Psychological safety is the prerequisite for any Just Culture, not its by-product. Regular, structured reflection formats, such as SEAs and M&M rounds, can help anchor collective learning processes within clinic work.

## 5 Limitations of this study

One important limitation of this study is that participation was voluntary. It is possible that veterinarians with strong opinions about the topic or a particular interest were more likely to take part. This suggests that the results may not fully represent all veterinarians, especially with regard to how common certain experiences or attitudes are. At the same time, such selective participation mainly affects representativeness, while patterns and associations observed within the participating group can still be meaningful (62). As the survey relied on participants’ memory and experiences, the responses may be subject to recall bias. In addition, most participants were women (over 90%). Although women make up the majority of the veterinary profession, this proportion is higher than average. According to the Veterinary Atlas of the Federal Chamber of Veterinarians, around 70% of veterinarians working in curative practice in 2024 were women (63). Women are also more likely to take part in questionnaire-based studies (64). This overrepresentation is important when interpreting the findings, as a survey on error reporting may reflect the views of those who are more willing to reflect on and report errors, while the perspectives of less engaged or more reluctant groups may be underrepresented (62). The survey was conducted in German and later translated into English for publication. Despite careful translation, small differences in how certain words or questions were understood cannot be completely ruled out. Such differences are unlikely to affect the overall findings but may influence the interpretation of individual questions.

## 6 Future research

The findings of this study give rise to several specific research questions. One important area concerns the emotional responses of veterinarians after clinical errors and how such experiences affect their psychological well-being, as well as strategies that may support coping in daily practice. Another focus is the implementation and evaluation of different error reporting systems to collect more objective data on errors and their contributing factors; however, challenges remain as reporting systems are often underused. In addition, further studies are needed to assess to what extent essential topics such as time management, working under pressure, and communication are addressed in veterinary education and how well graduates feel prepared to manage these aspects in clinical practice. These avenues for future research may help to develop a more comprehensive and practice-oriented error culture in veterinary medicine.

## 7 Conclusion

This study provides the first systematic examination of veterinary error culture in German-speaking countries and shows that errors are frequent and structurally shaped. A key finding is that error perception is role-dependent, meaning that no single professional perspective captures the full error landscape. At the same time, many institutions lack the structural conditions required for systematic learning.

Taken together, these results indicate that the future of veterinary patient safety depends less on eliminating individual mistakes than on strengthening organizational learning. This requires reliable leadership behavior, psychologically safe environments, and reporting pathways that are transparent, anonymous and non-punitive. Equally, non-technical skills such as communication, time prioritization and reflective teamwork must become integral components of veterinary education and professional development.

Ultimately, veterinary practices do not improve by denying the existence of errors, but by engaging with them constructively. Institutions that succeed in viewing errors as systemic learning opportunity – rather than individual failings – create the foundations for safer patient care, more sustainable working conditions and resilient teams.

## Data Availability

The raw data supporting the conclusions of this article will be made available by the authors, without undue reservation.
