# Supplementary material for: Understanding error culture in veterinary medicine: a survey among veterinarians across German-speaking countries
Source: Front Vet Sci. 2026 Apr 7;13:1784869. doi: 10.3389/fvets.2026.1784869 (PMC13097606; doi:10.3389/fvets.2026.1784869)
Supplement: Supplementary file 1 [file Table_1.docx]

| **Question Group** | ***Survey question and answer*** | **Question Type** |
| --- | --- | --- |
| ***Professional Background*** | | |
|  | When did you receive your veterinary licence?   - <5 years - 6-10 years - 11-15 years - >15 years | Single choice |
|  | Do you hold a supervisory or managerial role?   - Yes - No - Don’t know | Single choice |
| ***If supervisory role*** | | |
|  | How many staff members are you responsible for as a supervisor?   - up to 10 employees - 11-20 employees - 21-50 employees - >50 employees | Single choice |
|  | How would you describe your approach to supervision?   - I check all tasks - I perform random checks of tasks - I do not check any tasks - I am available for questions | Multiple choice |
| ***Received Errors (Supervisors only)*** | | |
|  | Have you ever received an error report that affected the health or welfare of an animal?   - Yes - No - Don’t remember | Single choice |
|  | In your opinion, in which areas did the reported errors most frequently occur?   - Patient history interview - Animal restraint - Handover of patients to colleagues - Diagnosis - Billing - Medication administration - Medication dosing - Surgery - Anaesthesia - Euthanasia - Interaction with the pet owner - Team interaction | Ranking scale |
|  | Which factors contributed to the reported errors?   - Inadequate equipment - Lack of experience - Lack of supervision - Lack of assistance (e.g. no veterinary nurse available) - Time pressure / lack of time - Hectic work environment - Fatigue - Team communication - Communication with the owner | Single choice |
|  | What do you believe are the reasons why no errors have been reported to you so far?   - No errors had occurred - Considered irrelevant - Concern about personal reputation - Concern about professional consequences - Concern about legal consequences - Concern about reactions to error reports (e.g. rejection, anger, etc.) - Other | Multiple choice |
| ***Self-Reported Errors*** | | |
|  | Has an animal under your care ever suffered permanent harm or died because of an incident?   - Yes - No - Don’t remember | Single choice |
|  | Have you ever reported an error that affected the health or welfare of an animal?   - Yes - No - Don’t remember | Single choice |
|  | In your opinion, in which tasks did the errors you reported most frequently occur?   - Patient history interview - Animal restraint - Handover of patients to colleagues - Diagnosis - Billing - Medication administration - Medication dosing - Surgery - Anaesthesia - Euthanasia - Interaction with the pet owner - Team interaction | Ranking scale |
|  | Which factors contributed to the occurrence of errors on your part?   - Inadequate equipment - Lack of experience - Lack of supervision - Lack of assistance (e.g. no veterinary nurse available) - Time pressure / lack of time - Hectic work environment - Fatigue - Communication | Single choice |
|  | What do you believe are the reasons why you have not reported any errors so far?   - No errors had occurred - Considered irrelevant - Concern about personal reputation - Concern about professional consequences - Concern about legal consequences - Concern about the emotional reaction to error reports in discussions with a supervisor - Concern about the emotional reaction to error reports in discussions with an owner - Other | Multiple choice |
|  | Who do you usually talk to when discussing your own errors?   - Colleagues - Supervisor - Uninvolved persons (friends/family) - Other | Multiple choice |
| ***Observed Errors*** | | |
|  | Have you ever observed an error report being made?   - Yes - No - Don’t remember | Single choice |
|  | In your opinion, in which tasks did the observed errors most frequently occur?   - Patient history interview - Animal restraint - Handover of patients to colleagues - Diagnosis - Billing - Medication administration - Medication dosing - Surgery - Anaesthesia - Euthanasia - Interaction with the pet owner - Team interaction | Ranking scale |
|  | Were you aware of the factors that contributed to the observed errors?   - Yes - No - Don’t remember | Single choice |
|  | Which factors contributed to the occurrence of the observed errors?   - Inadequate equipment - Lack of experience - Lack of supervision - Lack of assistance (e.g. no veterinary nurse available) - Time pressure / lack of time - Hectic work environment - Fatigue - Team communication - Communication with the owner | Single choice |
| ***Workplace Information*** | | |
|  | How many staff members are employed at your facility?   - <10 - 11-20 - 21-50 - >51 - Unknown | Single choice |
|  | How many patients do you treat on an average working day?   - <10 patients - 11-20 patients - 21-30 patients - >31 patients - Unknown | Single choice |
|  | What is your current professional position?   - General Practitioners - Intern - Doctoral Candidates - Veterinarians in Training - Specialist Veterinarians - Senior Veterinarians - Other | Multiple choice |
|  | What type of facility do you primarily work in?   - Practice - Clinic - Large Veterinary Employers (e.g. AniCura/Evidensia) - Academic Institution - Other | Multiple choice |
|  | What is your employment status?   - Employee - Partner - Owner | Single choice |
| ***Current Error Culture*** | | |
|  | How are causes of errors typically assessed in your workplace?   - Systemic error ("the system is at fault") - Individual error ("the person is at fault") - Systemic and individual error - Cause of error not clearly identified - Unknown | Single choice |
|  | Does your facility have an error reporting system?   - No error reporting system - Anonymous error reporting system - Designated contact person - Unknown | Single choice |
|  | How are errors usually discussed in your facility?   - In extraordinary team meetings (e.g. Morbidity and Mortality rounds) - In regular team meetings - Individually (one-to-one conversations) - Staff from quality management - Other | Single choice |
|  | How would you assess the following aspects in your facility? Tick whether missing, adequate, or needs improvement:   - The error reporting system? - The time – to adequately respond to the needs of your patients and to prevent future errors? - The resources – to adequately respond to the needs of your patients and to prevent future errors? - The level of support from supervisors? - The level of support from veterinary nurses/assistants? - The level of moral support? | Matrix |
| ***Demographics*** | | |
|  | What year were you born?   - XXXX | Year |
|  | What is your gender identity?   - Female - Male - Diverse / non-binary - No response | Single choice |
| ***Open Question*** | | |
|  | Is there anything else you would like to share with us on this topic? | Free-text response |

All survey questions were translated into English. Some German terms, however, are not directly transferable; for example, “veterinarian in training” was used to approximate the German concept of „Tierarzt in Fachtierarztausbildung“ (specialist veterinary training).
